# Supplementary material for: A CD8+ NK cell transcriptomic signature associated with clinical outcome in relapsing remitting multiple sclerosis
Source: Nat Commun. 2021 Jan 27;12:635. doi: 10.1038/s41467-020-20594-2 (PMC7840761; doi:10.1038/s41467-020-20594-2)
Supplement: Supplementary file 1 — Supplementary Information [file 41467_2020_20594_MOESM1_ESM.pdf]

Supplementary Figure1

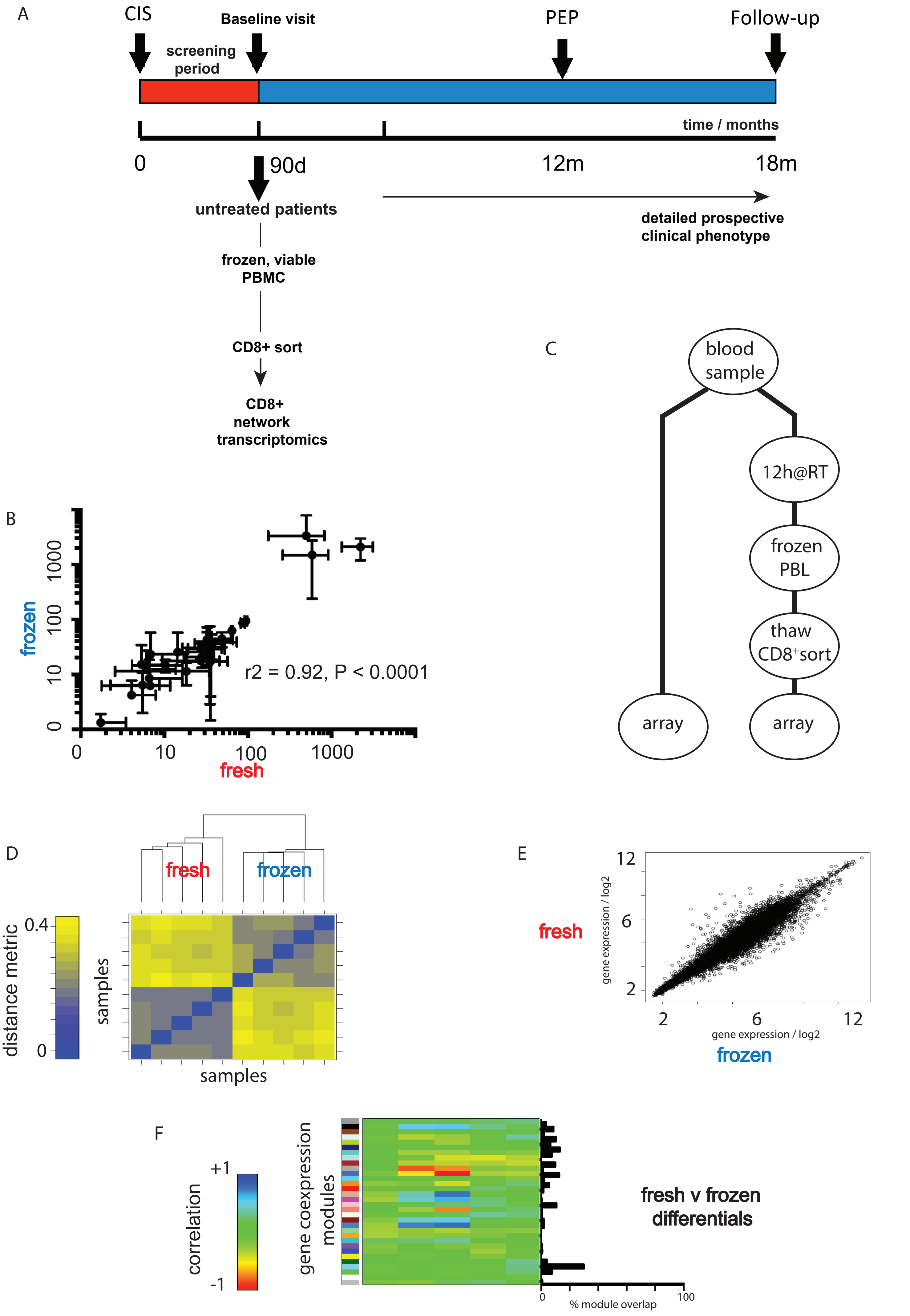

Supplementary Fig 1: Overview of the STAyCIS trial and sample processing

(A) Schematic timeline (days, d; months, m) illustrating STAyCIS trial endpoints and design. CIS = clinically isolated syndrome/1st demyelinating event, PEP = primary endpoint. Frozen, viable lymphocytes were collected as a PBMC (peripheral blood mononuclear cell) fraction at baseline enrolment (within 90 days of CIS) and frozen in liquid nitrogen. On thawing, a CD8^{+} subfraction was isolated by magnetic bead enrichment and analysed by microarray as described, comparing to detailed longitudinal clinical phenotyping (details available at www.itntrialshare.org). (B) Scatterplot showing fresh v frozen immunophenotyping, including major cell subset proportions and selected surface marker MFI for 27 immune cell subsets and traits when processed fresh (x-axis) or following freeze/thaw cycling (y-axis), values represent mean +/- SEM, n=5. Spearman correlation (r2) from linear regression with two-tailed P-value (C) Schematic outline of test protocol for fresh/frozen transcriptomic comparison. (D) Heatmap of distance matrix illustrating distinction between samples processed fresh or frozen as in C. (E) Scatterplot illustrating transcriptional differences between CD8^{+} enriched samples processed fresh (y-axis) and frozen (x-axis) as in (C), prior to transcriptomic analysis. (F) Heatmap illustrating mapping of fresh v frozen differentials as in (E) onto the transcriptomic network used (as in Fig 1A) to associate with clinical traits in the STAyCIS study.

Supplementary Figure 2

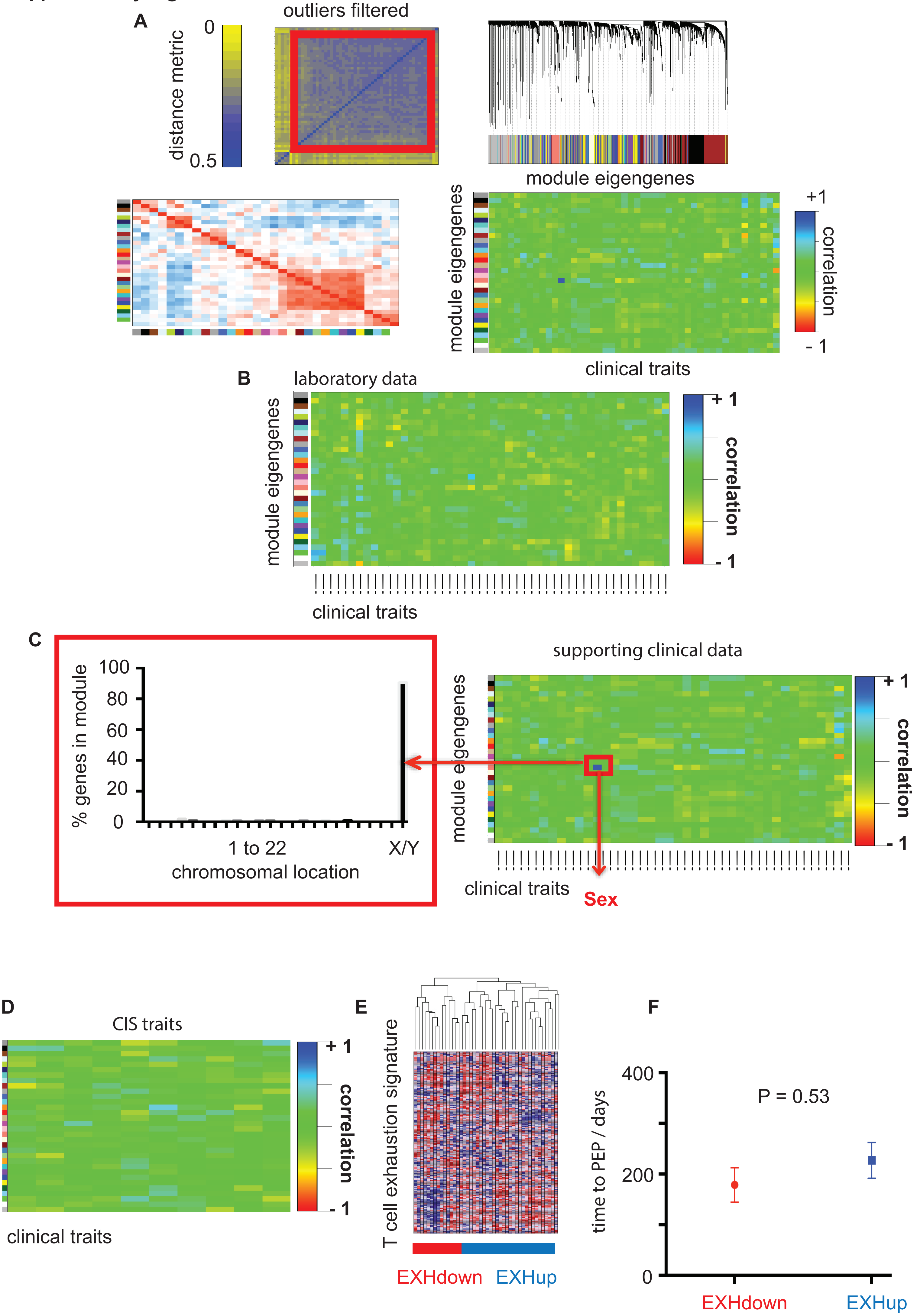

Supp Fig 2: Network analysis of STAyCIS and accessory data

(A) Schematic illustration of weighted gene coexpression network analysis with the STAyCIS dataset including QC filtering (top left), module identification and correlation with clinical traits (heatmap, bottom right). Each coloured block represents a distinct module of coexpressed genes identified by the network. (B) Heatmap illustrating correlation of module eigengenes with laboratory data (ordered traits listed in Supp Table 1). (C) Heatmap illustrating correlation of supporting clinical data with modular eigengenes. Strong, significant correlation was apparent between gender and a module that almost exclusively comprised X/Y-linked transcript expression (red box). (D) Heatmap illustrating correlation of modular eigengenes (y, coloured blocks) with baseline CIS traits (E) Heatmap illustrating unsupervised hierarchical clustering of the STAyCIS $CD8^+$ transcriptional dataset by the T cell exhaustion signature previously shown to correlate with clinical outcome in multiple autoinflammatory diseases. (F) Time to primary endpoint for MS patient subgroups defined by the T cell exhaustion signature as indicated in panel (E). Mann –Whitney two-tailed test of significance, P = 0.53. PEP = primary endpoint, EXH = exhaustion. For traits included in panels B-D see Supplementary Table 1.

Supplementary Figure 3

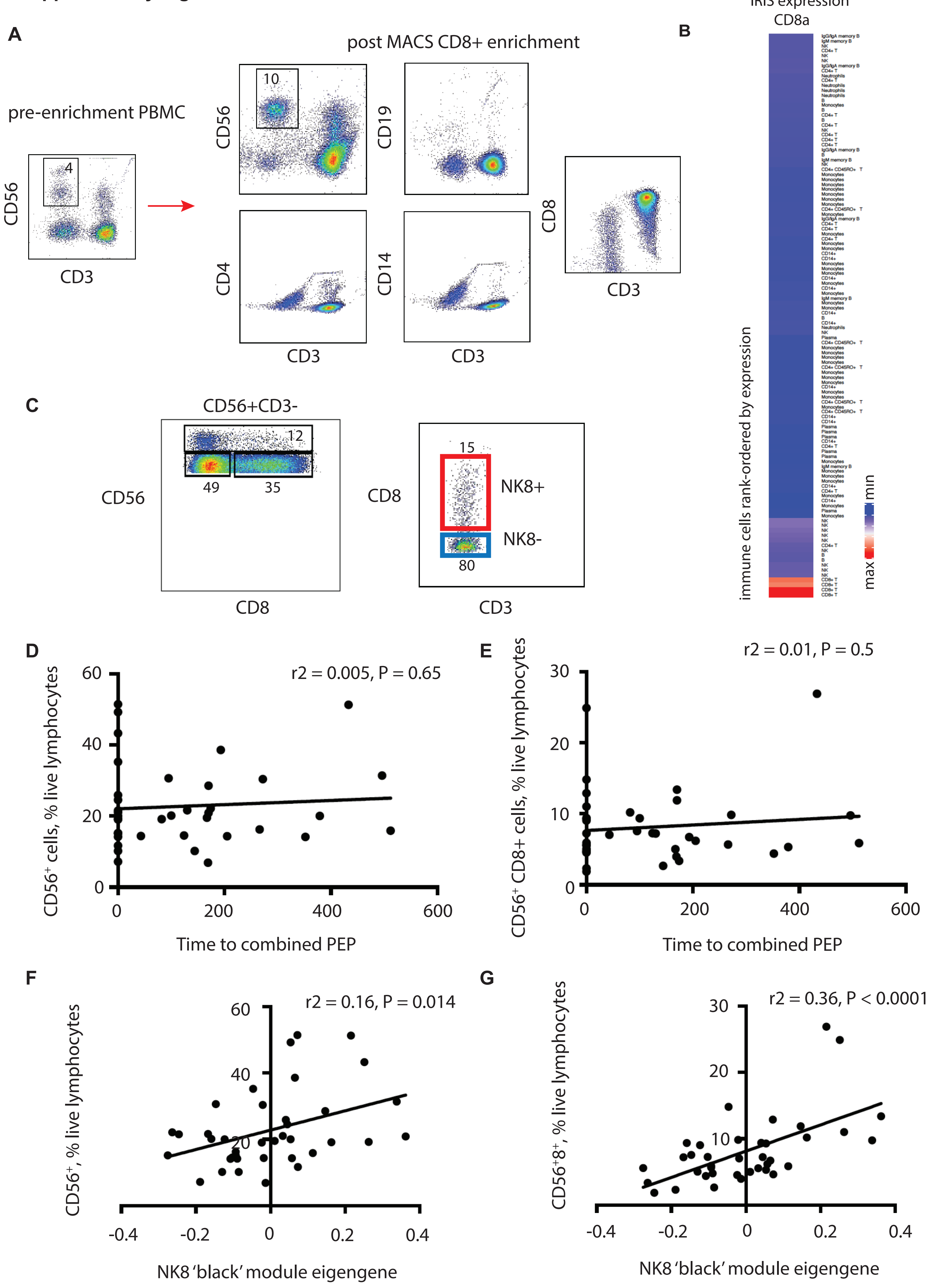

Supp Fig 3: in vitro NK functional studies

(A) Representative pseudocolored scatterplots illustrating MACS enrichment of $CD8^+$ cells with $CD3^-CD56^+CD8^+$ cells as the principal non-T cell 'contaminant' population. Post enrichment plots show a representative sample gated by major cell surface markers. (B) Heatmap illustrating mRNA expression of CD8 by immune cell subtype. Y-axis ranked by CD8 expression level across all immune cell types in immune response in silico (IRIS) dataset (C) Representative pseudocolored scatterplots illustrating CD8 expression on $CD56^{bright}$ and $CD56^{dim}$ natural killer cell subsets. (D-G) Scatterplots illustrating correlation (linear regression with Pearson r^2 and two-tailed significance test) of $\%CD56^+$ total (D,F % lymphocytes) and $\%CD56^+CD8^+$ (E,G % lymphocytes) with clinical outcome directly (D, E) and with the $NK8^+$ 'black' module eigengene (F,G).

Supplementary Figure 4

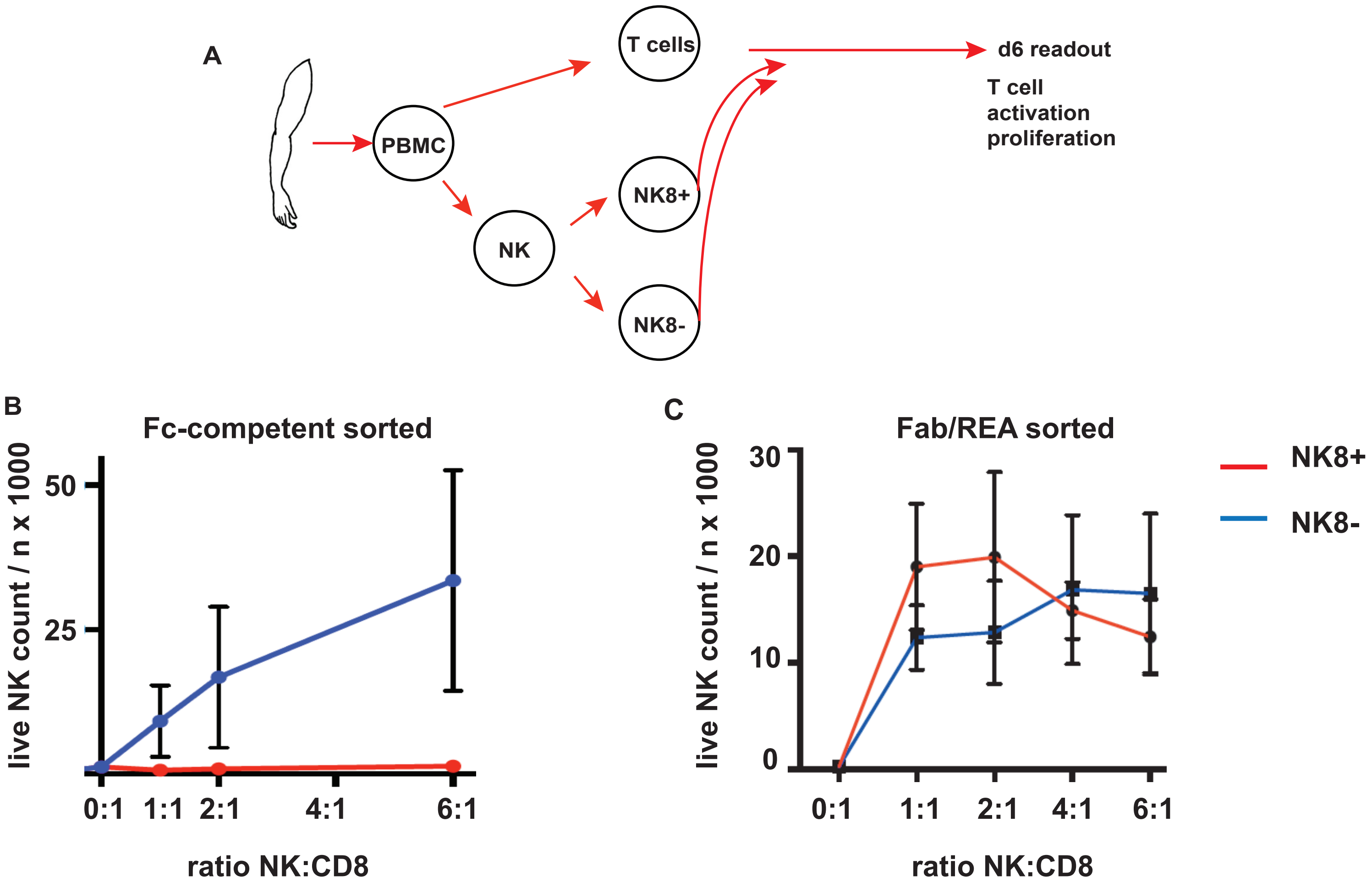

Supp Fig 4: NK/T cell co-culture

(A) Schematic illustration of autologous T cell and $NK8^{+/-}$ co-culture experiment. (B, C) Line and scatterplots illustrating $NK8^{+/-}$ survival during titrated coculture with autologous T cells following NK sorting with Fc-competent antibodies (B) or either Fc-incompetent antibodies/Fab fragments. Sorting NK with Fc-competent antibodies resulted in substantial and differential fratricide of $NK8^{+}$ cells, due to retention of $CD8^{+}$ antibodies and resultant Fc-mediated cell activation. This was not seen during functional studies reported here, which were performed using Fab and recombinantly engineered Fc-binding deficient antibodies (as in C). For B, C: n = 3, 10 independent replicates respectively; error bars = mean +/- sem.

Supplementary Figure 5

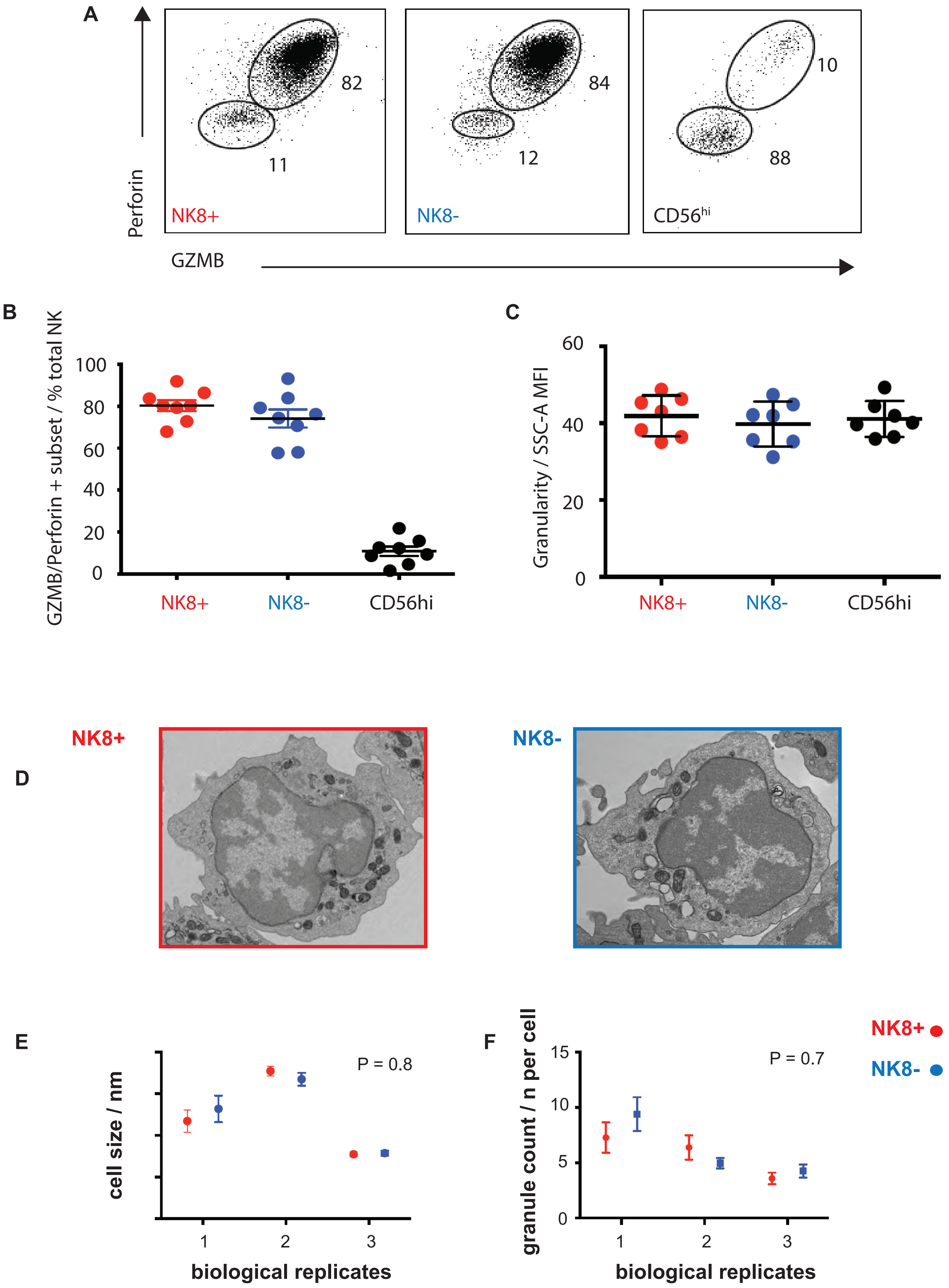

Supp Fig 5: Cytotoxic potential of NK subsets

(A-C) Representative flow cytometry scatterplots (A) and summary scatterplots (B, C) illustrating cytotoxic granule protein (perforin, y axis; granzyme B, x-axis) expression and cell granularity (SSC-A MFI) in $NK8^+$ (red), $NK8^-$ (blue) and $CD56^{hi}$ (black) NK subsets. For B, C: n = 8, 7 independent biological replicates respectively (D) Representative transmission electron microscopy (TEM) images of $NK8^+$ (left, red) and $NK8^-$ (right, blue) cells. (E, F) Scatterplots summarising quantification of cell size (E) number of lytic granules per cell (F) for $NK8^+$ (red) and $NK8^-$ (blue). Mean +/- SEM for 10 TEM images from flow sorted purified NK subsets across three biological replicates (from healthy donors). P = two-tailed Mann-Whitney test.

Supplementary Figure 6

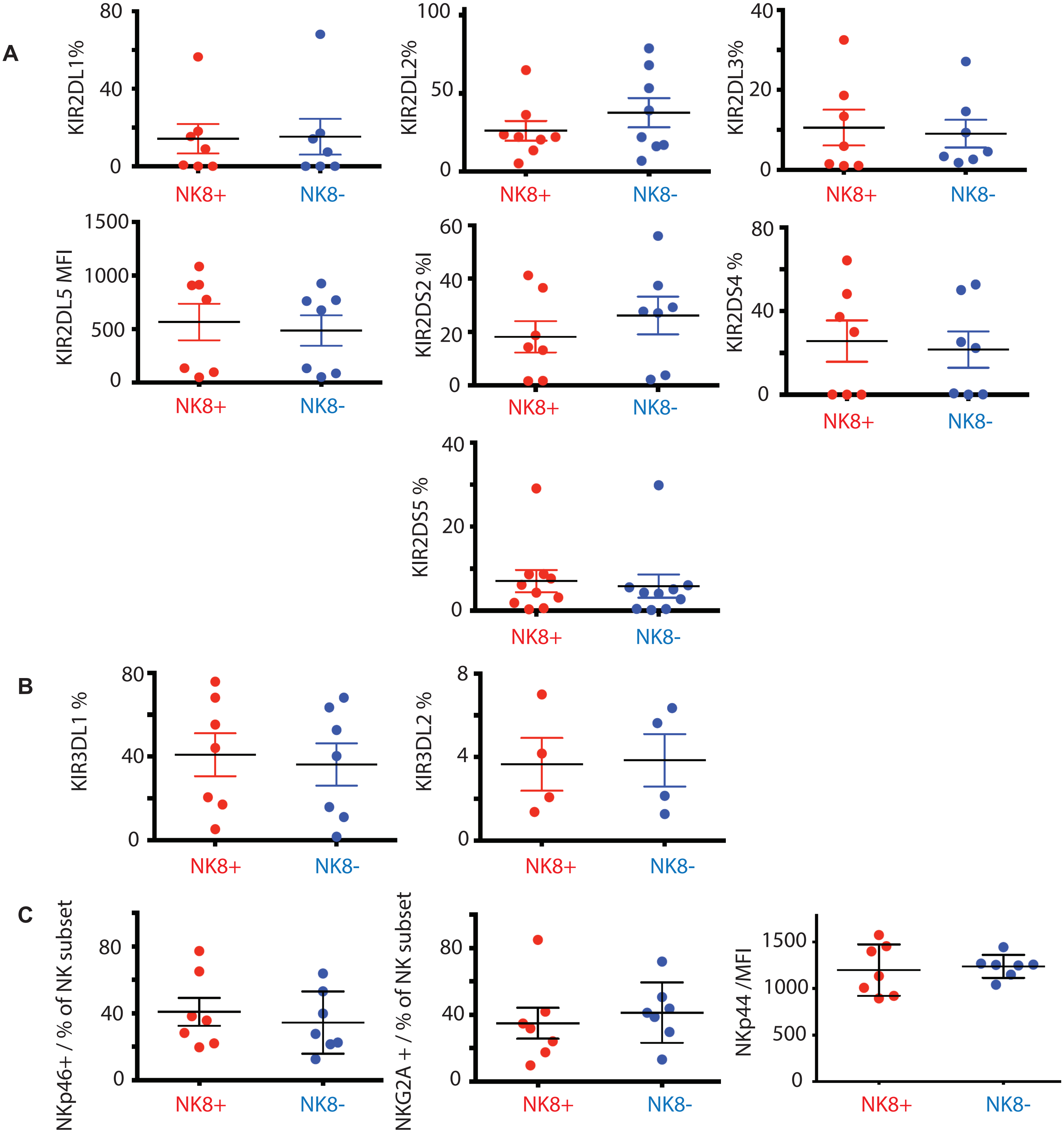

Supp Fig 6: Activating/inhibitory NK cell receptor expression by NK8$^+$ and NK8$^-$ subsets
Scatterplots illustrating comparison of NK cell activating/inhibitory receptor protein expression on NK8$^+$ and NK8$^-$ cells measured by flow cytometry using a validated panel of KIR-targeting antibodies (Suppl Table 4). (A) KIR2 family expression, (B) KIR3 receptor expression, (C) selected other receptors. For A: n=7-10 replicates as indicated, B: n=4-7 and C = 7 independent biological replicates.

Supplementary Figure 7

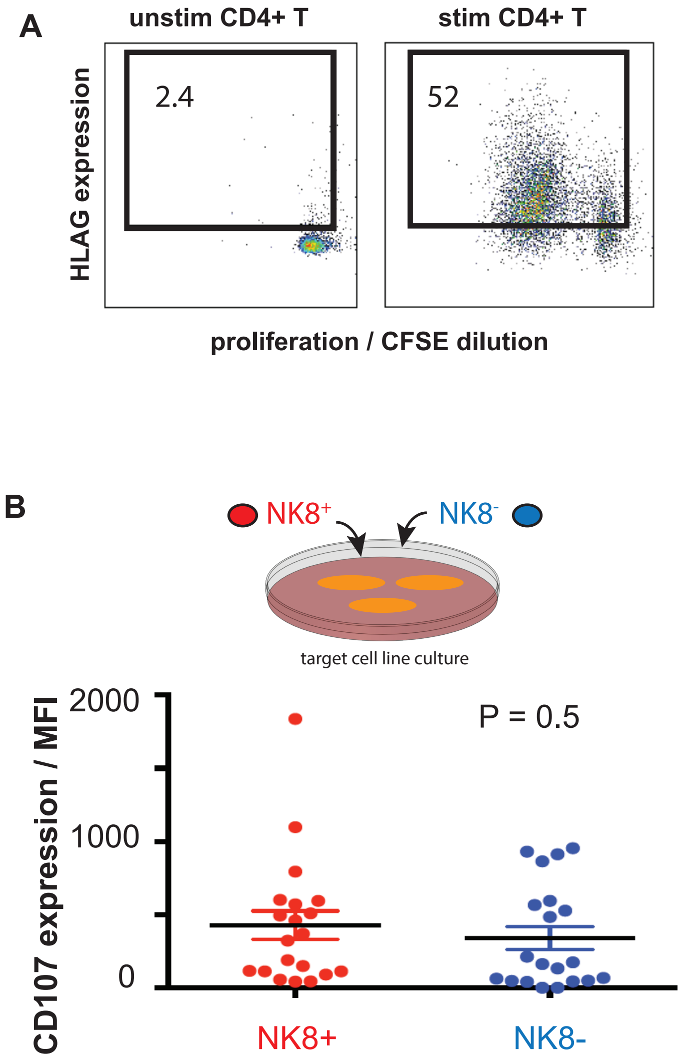

Supp Fig 7: HLA-G expression on CD4 cells and induced cytotoxicity of NK subsets

(A) Representative scatterplots illustrating HLA-G expression (y-axis) on unstimulated (left) and polyclonally stimulated $CD4^+$ T cells (right). x-axis = CFSE dilution reflecting proliferation. (B) Scatterplot showing induced NK cytotoxicity (CD107 MFI, y-axis) on coculture of $NK8^+$ (red) or $NK8^-$ (blue) with tumour cell lines (K562/Jurkat). For B, n=20 independent biological replicates; error bars = mean +/- sem with Mann-Whitney two-tailed test of significance.

Supplementary Figure 8

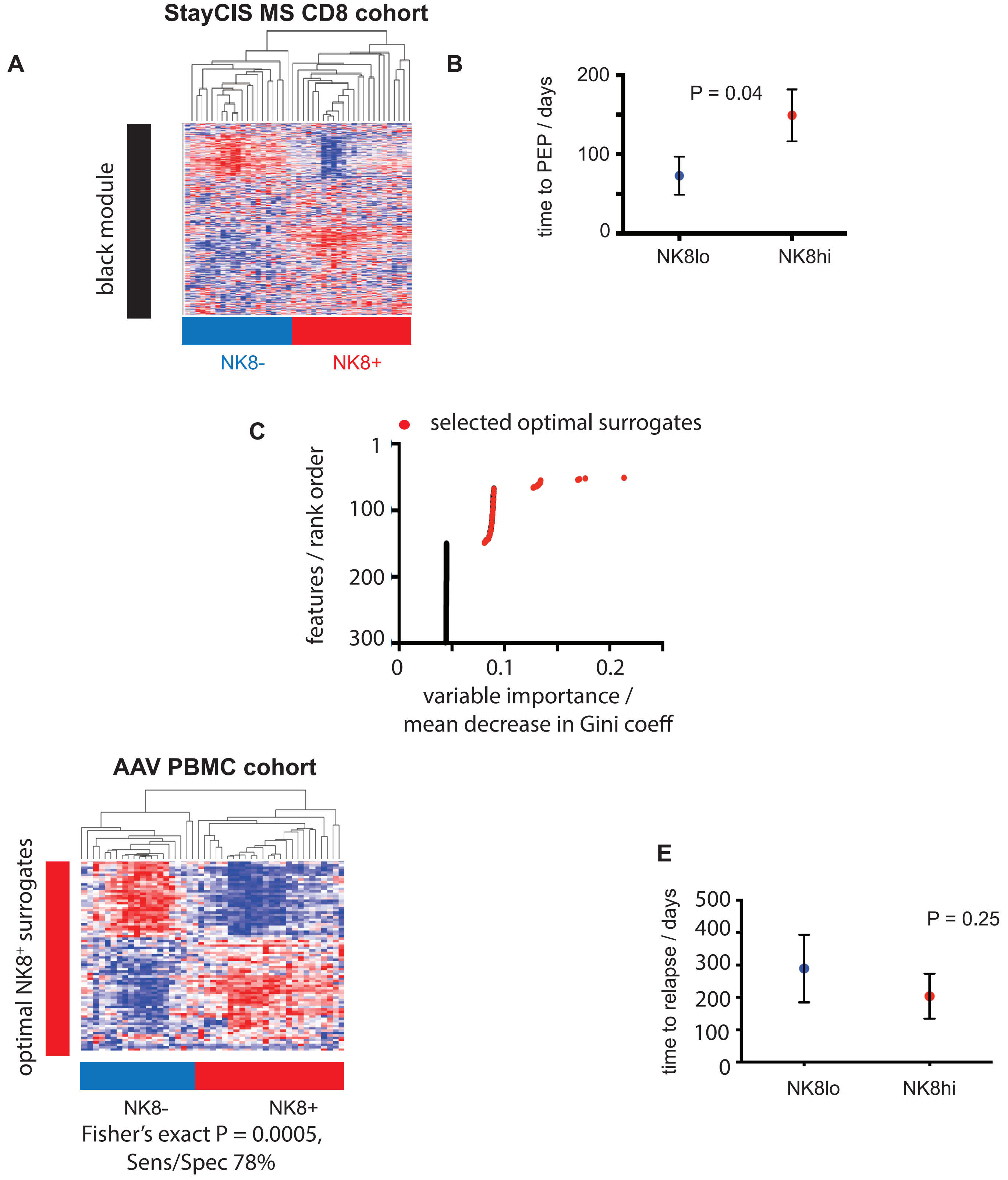

Supp Fig 8. Identification of surrogate markers of an NK8$^+$ signature in PBMC samples for validation purposes

Heatmap illustrating hierarchical clustering of the STAyCIS MS cohort using the 'black' module genes (A) with associated clinical outcome of identified patient subgroups (B) as in Fig 1C. (C) Scatterplot showing ranked optimal predictive genes (y-axis) against feature importance (x-axis, decrease in Gini Coefficient for models not incorporating each feature) following randomforests feature selection identification, searching for analogous subgroups in a matched PBMC cohort of anti-neutrophil antibody associated vasculitis (AAV) patients. This set of features was then used for clustering of an independent MS cohort as shown in Fig 3D. (D) Heatmap illustrating hierarachical clustering of PBMC data from n=47 patients (columns) with AAV using the optimal surrogate markers of the MS black eigengene (rows). (E) Dot plot showing NK8lo and NK8hi subgroups from AAV cohort in panel D and associated time to relapse (mean +/- sem).

Supplementary Figure 9

A

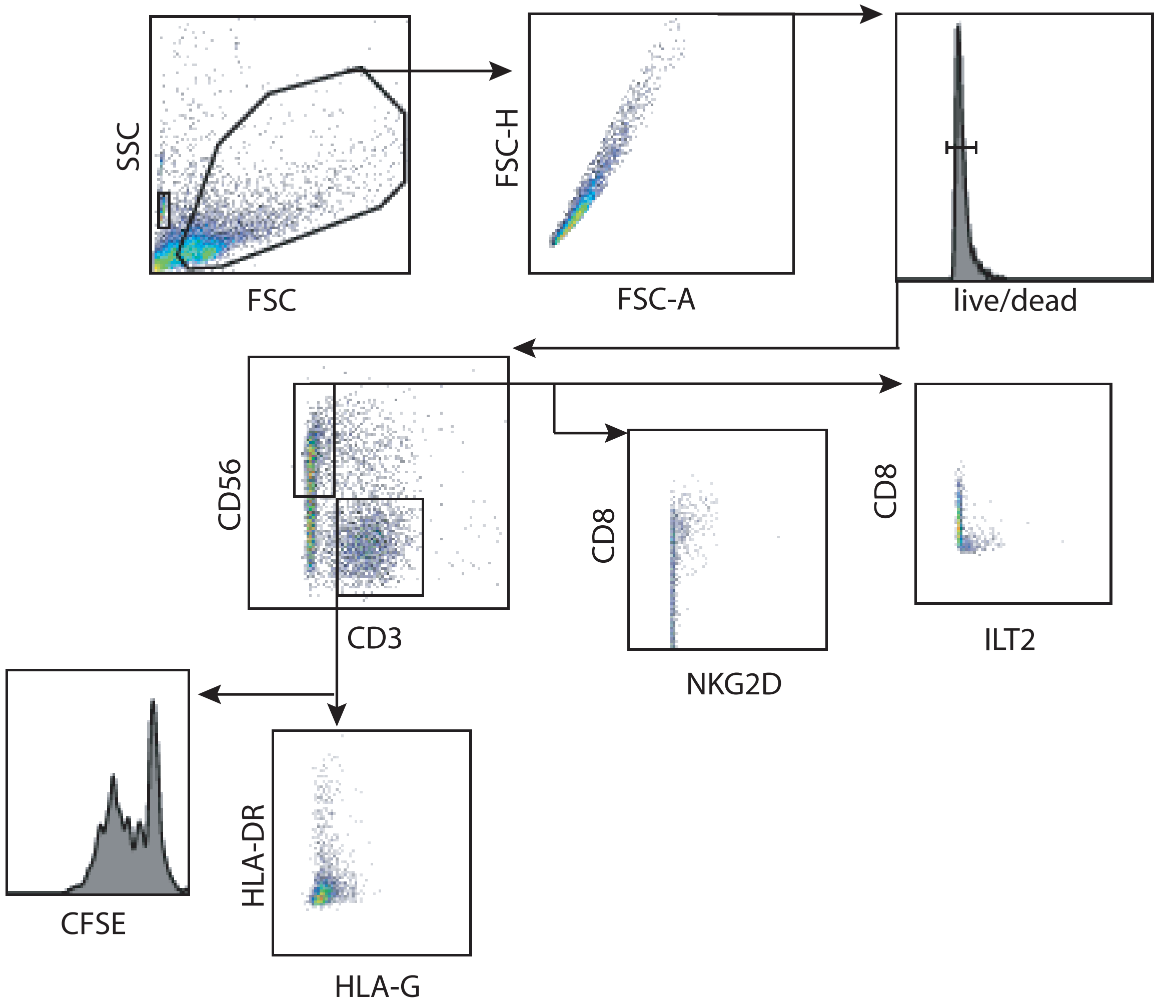

B

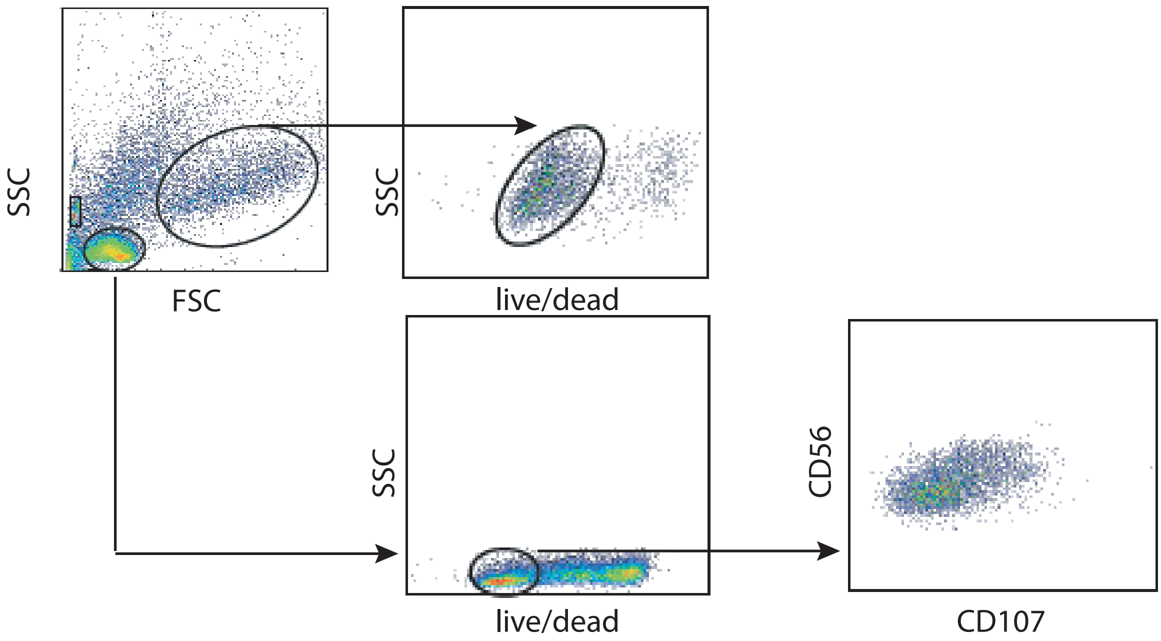

Supp Fig 9. Flow cytometry gating strategies

Representative scatterplots showing flow cytometry gating strategies for (A) NK immunophenotyping (relating to Fig2, Supp Figs4-6) and (B) NK cytotoxicity assays (relating to Fig3, Supp Fig7).
