# Supplementary material for: A CD8+ NK cell transcriptomic signature associated with clinical outcome in relapsing remitting multiple sclerosis
Source: Nat Commun. 2021 Jan 27;12:635. doi: 10.1038/s41467-020-20594-2 (PMC7840761; doi:10.1038/s41467-020-20594-2)
Supplement: Supplementary file 3 — Reporting Summary [file 41467_2020_20594_MOESM3_ESM.pdf]

nature research

Corresponding author(s): NCOMMS-19-8110828B
Last updated by author(s): 29/09/2020

Reporting Summary

Nature Research wishes to improve the reproducibility of the work that we publish. This form provides structure for consistency and transparency in reporting. For further information on Nature Research policies, see our Editorial Policies and the Editorial Policy Checklist.

Statistics

For all statistical analyses, confirm that the following items are present in the figure legend, table legend, main text, or Methods section.

n/a	Confirmed	
☐	☒	The exact sample size (*n*) for each experimental group/condition, given as a discrete number and unit of measurement
☐	☒	A statement on whether measurements were taken from distinct samples or whether the same sample was measured repeatedly
☐	☒	The statistical test(s) used AND whether they are one- or two-sided<br>*Only common tests should be described solely by name; describe more complex techniques in the Methods section.*
☐	☒	A description of all covariates tested
☐	☒	A description of any assumptions or corrections, such as tests of normality and adjustment for multiple comparisons
☐	☒	A full description of the statistical parameters including central tendency (e.g. means) or other basic estimates (e.g. regression coefficient) AND variation (e.g. standard deviation) or associated estimates of uncertainty (e.g. confidence intervals)
☐	☒	For null hypothesis testing, the test statistic (e.g. *F*, *t*, *r*) with confidence intervals, effect sizes, degrees of freedom and *P* value noted<br>*Give P values as exact values whenever suitable.*
☒	☐	For Bayesian analysis, information on the choice of priors and Markov chain Monte Carlo settings
☒	☐	For hierarchical and complex designs, identification of the appropriate level for tests and full reporting of outcomes
☐	☒	Estimates of effect sizes (e.g. Cohen's *d*, Pearson's *r*), indicating how they were calculated

Our web collection on statistics for biologists contains articles on many of the points above.

Software and code

Policy information about availability of computer code

Data collection	All data were collected and analysed using R version 3.6.0 (2019-04-26) in combination with open source software as specified, freely available from Bioconductor version 3.11 or the comprehensive R archive network (CRAN).
Data analysis	All data were collected and analysed using R version 3.6.0 (2019-04-26) in combination with open source software as specified, freely available from Bioconductor version 3.11 or the comprehensive R archive network (CRAN).<br>Sequences were processed, cleaned and aligned using a combination of bbsplit, hisat2 and trimgalore with count data read into R using the subRead package (Bioconductor). Normalization and transformation were undertaken using edgeR and voom packages for Bioconductor in R with pairwise differential expression analysis (FDR threshold 5%) using edgeR.<br>Data QC was ensured using the arrayQualitymetrics (microarray) and fastqc, qorts and pcaexplorer (RNAseq) packages for Bioconductor in R.<br>Unsupervised identification of co-correlated genes was undertaken using the Weighted Gene Coexpression Network Analysis Bioconductor package in R. Hierarchical clustering was performed using a Pearson correlation distance metric and average linkage analysis performed using Genepattern.<br>Modular signatures were compared against cell and tissue-specific signatures as specified using Enrichr or fgsea (Bioconductor) with a significance threshold of 5% FDR.<br>Flow cytometry gating and population analysis were analyzed using WinList's™ (http://www.vsh.com) FCOM function or using manual gating as illustrated (Supp Fig 9).<br>For custom oligo spotted microarray data, normalised, log- transformed expression data was analysed using the ML Interfaces Bioconductor package in R.<br>For publicly-available validation, data was downloaded from GEO and imported into R using the Bioconductor package GEOquery in R.

For manuscripts utilizing custom algorithms or software that are central to the research but not yet described in published literature, software must be made available to editors and reviewers. We strongly encourage code deposition in a community repository (e.g. GitHub). See the Nature Research guidelines for submitting code & software for further information.

Data

Policy information about availability of data

All manuscripts must include a data availability statement. This statement should provide the following information, where applicable:

- Accession codes, unique identifiers, or web links for publicly available datasets
- A list of figures that have associated raw data
- A description of any restrictions on data availability

The datasets generated during and/or analysed during the current study areavailable in the ArrayExpress repository at the European Bioinformatics Institute, accession number E-MTAB-9637.
Clinical Trial data is available through the Immune Tolerance Network TrialShare Platform (https://www.itntrialshare.org).
Cell and tissue-specific transcripts from the Human Gene Atlas (HGA) are accessible through GEO (accession number GSE1133) and the Immune Response In Silico (IRIS) dataset is publicly-available through GEO (accession number GSE22886)

Field-specific reporting

Please select the one below that is the best fit for your research. If you are not sure, read the appropriate sections before making your selection.

☒ Life sciences ☐ Behavioural & social sciences ☐ Ecological, evolutionary & environmental sciences

For a reference copy of the document with all sections, see nature.com/documents/nr-reporting-summary-flat.pdf

Life sciences study design

All studies must disclose on these points even when the disclosure is negative.

Sample size	Sample size was determined as for the clinical study which served as the foundation for the current investigation and is described here: Neurology. 2012 Apr 10; 78(15): 1171–1178.
Data exclusions	Samples for gene expression profiling were only excluded if they failed technical QC as described in the methods and outlined in Supplementary Material. Exclusion criteria were on the basis of established, pre-specificed QC metrics obtained using the ArrayQualityMetrics package in R.
Replication	An independent, publicly-available validation cohort was used to validate the study's principal findings as described in the manuscript. Validation was only attempted on this single cohort as no other appropriate cohort was available.<br>For flow cytometry experiments and functional assays the number of independent replicates is as indicated in the figures with all data shown.
Randomization	Subject randomization was performed as for the clinical study which served as the foundation for the current investigation and is described here: Neurology. 2012 Apr 10; 78(15): 1171–1178.<br>For in vitro experiments subjects were selected in a blinded, random fashion by the collection of leucocyte cones from blood donors attending for donation.
Blinding	Blinding to clinical treatment group was undertaken as for the clinical study which served as the foundation for the current investigation and is described here: Neurology. 2012 Apr 10; 78(15): 1171–1178. Processing of the transcriptomic data was undertaken by investigators who were blinded to the results of the clinical study until transcriptional networks were defined.<br>For in vitro studies, investigators undertaking analysis were blinded to the source of samples and the treatments given until gates and expression values were determined.

Reporting for specific materials, systems and methods

We require information from authors about some types of materials, experimental systems and methods used in many studies. Here, indicate whether each material, system or method listed is relevant to your study. If you are not sure if a list item applies to your research, read the appropriate section before selecting a response.

Materials & experimental systems

n/a	Involved in the study	
☐	☒	Antibodies
☐	☒	Eukaryotic cell lines
☒	☐	Palaeontology and archaeology
☒	☐	Animals and other organisms
☐	☒	Human research participants
☐	☒	Clinical data
☒	☐	Dual use research of concern

Methods

n/a	Involved in the study	
☒	☐	ChIP-seq
☐	☒	Flow cytometry
☒	☐	MRI-based neuroimaging

Antibodies

Antibodies used

A complex but validated panel of antibodies were used for KIR typing with full description of the clone ids and antibodies provided in Supplementary Table 4.
anti-CD56 (Clone REA196), Miltenyi Biotech
anti-CD8a Fab (Clone BW135/80 Fab)
anti-CD3 REA (Clone REA641)
anti-HLA-G (Biolegend, clone 87G)
Granzyme B (Biolegend, clone GB11)
Perforin (eBioscience, eBioOMAK-D)
Live/Dead Blue reactive dye (Life Technologies)
KIR2D (Miltenyi, Clone NKVFS1)
KIR2DL1 (Clone 143211, Clone EB6B)
KIR2DL2 (Clones DX27, 180701, 5.133)
KIR2DL3 (Clone 180701)
KRI2DL4 (Clone mAb33)
KIR2DL5A/B (Clone UP-R1)
KIR2DS1 (Clone 413211, EB6B)
KIR2DS2 (Clone DX27, 180701, 5.133)
KIR2DS3 (JJC11.6,FES172)
KIR2DS4 (FES172)
KIR2DS5 (Clone 413211, 5.133)
KIR3DL1 (Clone DX9)
KIR3DL2 (Clone 5.133,Z27, 413211, FES172, DX27)
KIR3DS1 (Clone DX9, Z27)

Validation

Validation of the KIR typing antibody panel was undertaken independently and is described in full here: Czaja, K., et al. A comprehensive analysis of the binding of anti-KIR antibodies to activating KIRs. Genes Immun 15, 33-37 (2014).
Validation of other antibodies is as described by the manufacturers at the locations specified below:
anti-CD56 (Clone REA196): https://www.miltenyibiotec.com/GB-en/products/cd56-antibody-anti-human-reafinity-rea196.html#gref
anti-CD8a Fab (Clone BW135/80 Fab): https://www.miltenyibiotec.com/GB-en/products/cd8-antibody-anti-human-bw135-80.html#gref
anti-CD3 REA (Clone REA641): https://www.miltenyibiotec.com/GB-en/products/cd3-antibody-anti-mouse-reafinity-rea641.html#gref
anti-HLA-G (Biolegend, clone 87G, https://www.biolegend.com/en-us/products/pe-anti-human-hla-g-antibody-5212)
Granzyme B (Biolegend, clone GB11, https://www.biolegend.com/en-us/products/fitc-anti-human-mouse-granzyme-b-antibody-6066)
Perforin (eBioscience, eBioOMAK-D, https://www.thermofisher.com/antibody/product/Perforin-Antibody-clone-eBioOMAK-D-Monoclonal/17-9392-80)
Live/Dead Blue reactive dye (Life Technologies, https://www.thermofisher.com/order/catalog/product/L23105?gclid=EAIaIQobChMIs_i-28KO7AIViuntCh3DHgD0EAAYASAAEgKYTfD_BwE&s_kwcid=AL!3652!3!358504026899!p!!g!!live%20dead%20blue&ef_id=EAIaIQobChMIs_i-28KO7AIViuntCh3DHgD0EAAYASAAEgKYTfD_BwE:G:s&s_kwcid=AL!3652!3!358504026899!p!!g!!live%20dead%20blue&cid=bid_pca_frg_r01_co_cp1359_pjt0000_bid00000_0se_gaw_bt_pur_con#/L23105?gclid=EAIaIQobChMIs_i-28KO7AIViuntCh3DHgD0EAAYASAAEgKYTfD_BwE&s_kwcid=AL!3652!3!358504026899!p!!g!!live%20dead%20blue&ef_id=EAIaIQobChMIs_i-28KO7AIViuntCh3DHgD0EAAYASAAEgKYTfD_BwE:G:s&cid=bid_pca_frg_r01_co_cp1359_pjt0000_bid00000_0se_gaw_bt_pur_con)

KIR typing antibodies used:
KIR2D
Clone NKVFS1, https://www.miltenyibiotec.com/GB-en/products/kir2d-antibody-anti-human-nkvfs1.html#gref
KIR2DL1
Clone 143211, https://www.rndsystems.com/products/human-kir2dl1-kir2ds5-alexa-fluor-488-conjugated-antibody-143211_fab1844g
Clone EB6B, https://www.beckman.com/reagents/coulter-flow-cytometry/antibodies-and-kits/single-color-antibodies/cd158a-h
KIR2DL2:
Clone DX27, https://www.biolegend.com/en-us/products/purified-anti-human-cd158b-kir2dl2-l3--nkat2-antibody-2271
Clone 180701, https://www.rndsystems.com/products/human-kir2dl3-cd158b2-fluorescein-conjugated-antibody-180701_fab2014f
Clone 5.133, https://www.miltenyibiotec.com/GB-en/products/cd158e-k-kir3dl1-dl2-antibody-anti-human-5-133.html#gref
KIR2DL3
Clone 180701, https://www.rndsystems.com/products/human-kir2dl3-cd158b2-fluorescein-conjugated-antibody-180701_fab2014f
KRI2DL4
Clone mAb33, https://www.biolegend.com/en-us/products/pe-anti-human-cd158d-kir2dl4-antibody-6382
KIR2DL5A/B
Clone UP-R1, https://www.biolegend.com/en-us/products/pe-anti-human-cd158f-kir2dl5-antibody-5809
KIR2DS1
Clone 143211, https://www.rndsystems.com/products/human-kir2dl1-kir2ds5-alexa-fluor-488-conjugated-antibody-143211_fab1844g
Clone EB6B, https://www.beckman.com/reagents/coulter-flow-cytometry/antibodies-and-kits/single-color-antibodies/cd158a-h
KIR2DS2

Clone DX27, https://www.biolegend.com/en-us/products/purified-anti-human-cd158b-kir2dl2-l3--nkat2-antibody-2271
Clone 180701, https://www.rndsystems.com/products/human-kir2dl3-cd158b2-fluorescein-conjugated-antibody-180701_fab2014f
Clone 5.133, https://www.miltenyibiotec.com/GB-en/products/cd158e-k-kir3dl1-dl2-antibody-anti-human-5-133.html#gref
KIR2DS3
JJC11.6, https://www.miltenyibiotec.com/GB-en/products/cd158i-kir2ds4-antibody-anti-human-jjc11-6.html#gref
FES172, https://www.mybeckman.uk/reagents/coulter-flow-cytometry/antibodies-and-kits/single-color-antibodies/cd158i/im3337
KIR2DS4
FES172, https://www.mybeckman.uk/reagents/coulter-flow-cytometry/antibodies-and-kits/single-color-antibodies/cd158i/im3337
KIR2DS5
Clone 143211, https://www.rndsystems.com/products/human-kir2dl1-kir2ds5-alexa-fluor-488-conjugated-antibody-143211_fab1844g
Clone 5.133, https://www.miltenyibiotec.com/GB-en/products/cd158e-k-kir3dl1-dl2-antibody-anti-human-5-133.html#gref
KIR3DL1
Clone DX9, https://www.bdbiosciences.com/us/applications/research/t-cell-immunology/regulatory-t-cells/surface-markers/human/purified-mouse-anti-human-nkb1-dx9/p/555964
KIR3DL2
Clone 5.133, https://www.miltenyibiotec.com/GB-en/products/cd158e-k-kir3dl1-dl2-antibody-anti-human-5-133.html#gref
Clone Z27, https://www.beckman.com/reagents/coulter-flow-cytometry/antibodies-and-kits/single-color-antibodies/cd158e1-12
Clone 143211, https://www.rndsystems.com/products/human-kir2dl1-kir2ds5-alexa-fluor-488-conjugated-antibody-143211_fab1844g
Clone FES172, https://www.mybeckman.uk/reagents/coulter-flow-cytometry/antibodies-and-kits/single-color-antibodies/cd158i/im3337
Clone DX27, https://www.biolegend.com/en-us/products/purified-anti-human-cd158b-kir2dl2-l3--nkat2-antibody-2271
KIR3DS1
Clone DX9, https://www.bdbiosciences.com/us/applications/research/t-cell-immunology/regulatory-t-cells/surface-markers/human/purified-mouse-anti-human-nkb1-dx9/p/555964
Clone Z27, https://www.beckman.com/reagents/coulter-flow-cytometry/antibodies-and-kits/single-color-antibodies/cd158e1-12

Eukaryotic cell lines

Policy information about cell lines

Cell line source(s)	Cell lines used were Jurkat and K562 lines and were gifts from collaborators within the host institution (Dr J Sowerby)
Authentication	Cell lines were authenticated by flow cytometry for characteristic surface markers.
Mycoplasma contamination	Routine mycoplasma testing was undertaken for all established cell lines used and were repeatedly negative throughout.
Commonly misidentified lines (See ICLAC register)	No commonly misidentified cell lines were used in the study.

Human research participants

Policy information about studies involving human research participants

Population characteristics	Population characteristics of human participants recruited into the clinical studies used are detailed in Supplementary Tables 1, 5, 6 and 7
Recruitment	Recruitment was undertaken as for the clinical study which served as the foundation for the current investigation and is described here: Neurology. 2012 Apr 10; 78(15): 1171–1178.
Ethics oversight	The trial was sponsored by NIAID in collaboration with the Immune Tolerance Network (clinicaltrials.gov NCT00094172). The study protocol and informed consent documents were approved by appropriate local ethics review committees and/or Institutional Review Boards. The study was approved by institutional review boards at 14 centers in the United States and Canada. Written informed consent was obtained from subjects prior to enrollment in the STAyCIS study (NCT00094172).

Note that full information on the approval of the study protocol must also be provided in the manuscript.

Clinical data

Policy information about clinical studies
All manuscripts should comply with the ICMJEguidelines for publication of clinical research and a completedCONSORT checklist must be included with all submissions.

Clinical trial registration	clinicaltrials.gov NCT00094172
Study protocol	The trial protocol can be obtained from clinicaltrials.gov (NCT00094172) and the founding clinical study has been published here: Neurology. 2012 Apr 10; 78(15): 1171–1178.
Data collection	Data collection was undertaken as for the clinical study which served as the foundation for the current investigation and is described here: Neurology. 2012 Apr 10; 78(15): 1171–1178.

Outcomes	Primary and secondary outcomes were defined as for the clinical study which served as the foundation for the current investigation and is described here: Neurology. 2012 Apr 10; 78(15): 1171–1178.

Flow Cytometry

Plots

Confirm that:

- [x] The axis labels state the marker and fluorochrome used (e.g. CD4-FITC).
- [x] The axis scales are clearly visible. Include numbers along axes only for bottom left plot of group (a 'group' is an analysis of identical markers).
- [x] All plots are contour plots with outliers or pseudocolor plots.
- [x] A numerical value for number of cells or percentage (with statistics) is provided.

Methodology

Sample preparation	Venous blood was obtained from participants and frozen as aliquots of 1x107 cells stored in liquid nitrogen as described in methods. Samples were thawed and processed immediately as described.
Instrument	Following staining, data were acquired on a FACSCanto flow cytometer (BD Biosciences)
Software	Data were analyzed using WinList's™ (http://www.vsh.com) FCOM function
Cell population abundance	Samples were purified using magnetic bead separation as described with purity confirmed by flow cytometry of pre- and post-sort fractions.
Gating strategy	The gating strategy adopted is outlined in Supplementary Figure 3A and C

- [x] Tick this box to confirm that a figure exemplifying the gating strategy is provided in the Supplementary Information.
