# Supplementary material for: A CD8+ NK cell transcriptomic signature associated with clinical outcome in relapsing remitting multiple sclerosis
Source: Nat Commun. 2021 Jan 27;12:635. doi: 10.1038/s41467-020-20594-2 (PMC7840761; doi:10.1038/s41467-020-20594-2)
Supplement: Supplementary file 4 — Description of Additional Supplementary Files [file 41467_2020_20594_MOESM4_ESM.pdf]

Descriptions of Additional Supplementary Files

Supplementary Data 1
Description: Association of NK8 'black' module with baseline laboratory data, supporting clinical data and baseline clinical traits (relating to heatmaps shown in Supplementary figs 2B, C and D respectively).

Supplementary Data 2
Description: NK enriched 'black' module feature description and annotation

Supplementary Data 3
Description: Genes differentially expressed by NK8+ and NK8- subsets

Supplementary Data 4
Description: Staining pattern and antibody clone ids for flow cytometric quantification of KIR receptors

Supplementary Data 5
Description: Optimal surrogate genes reflecting the NK8+ subgroups in PBMC samples

Supplementary Data 6
Description: Clinical characteristics of AAV cohort.

Supplementary Data 7
Description: Clinical Characteristics of MS Validation cohort by prognostic subgroup
